# Lactoferrin Solution as a New Natural Photosensitizer in Photodynamic Therapy Against Oral *Candida* spp. Multidrug-Resistant Isolates: A Preliminary In Vitro Study

**DOI:** 10.3390/microorganisms13061255

**Published:** 2025-05-29

**Authors:** Cinzia Casu, Andrea Butera, Alice Piga, Andrea Scribante, Sara Fais, Germano Orrù

**Affiliations:** 1 Oral Biotechnology Laboratory, Department of Surgical Science, University of Cagliari, 09124 Cagliari, Italy; cinzia.casu2@unica.it (C.C.); ali.piga87@gmail.com (A.P.); sara.fais@unica.it (S.F.); germano.orru@unica.it (G.O.); 2 Unit of Dental Hygiene, Section of Dentistry, Department of Clinical, Surgical, Diagnostic and Pediatric Sciences, University of Pavia, 27100 Pavia, Italy; 3 Unit of Orthodontics and Pediatric Dentistry, Section of Dentistry, Department of Clinical, Surgical, Diagnostic and Pediatric Sciences, University of Pavia, 27100 Pavia, Italy

**Keywords:** lactoferrin, photodynamic therapy, candida, in vitro study

## Abstract

Serious oral infections are frequently caused by Candida species, which have lately demonstrated resistance to antifungal medications. As a result, new therapeutic strategies, like photodynamic therapy (PDT), are desperately needed. Lactoferrin (LF), a salivary enzyme, is a natural protein that binds iron and has antifungal properties. Given its chemical structure and light absorption at 310–350 nm, LF appears to be a good photosensitizer in a PDT process for treating oral candidiasis. The purpose of this work was to assess the effectiveness of lactoferrin (LF) as a photosensitizer (PS) in photodynamic treatment (PDT) against oral multidrug-resistant (MDR) isolates of *Candida* spp. using an in vitro investigation. For this in vitro investigation, oral MDR isolates of *Candida albicans*, *Candida kruseii*, and *Candida glabrata* were employed. Using a Kirby–Bauer test (Eucast protocol), a solution of 20 mg of bovine lactoferrin dissolved in 1 mL of Sabouraud’s broth was tested in four different experimental combinations: (i) the solution as it is; (ii) the solution activated with 3% H_2_O_2_; (iii) the solution activated by light at 310–350 nm; and (iv) the solution activated with both 3% H_2_O_2_ and light at 310–350 nm. A control group and one with only H_2_O_2_ were also tested. After that, the Petri plates were incubated for 48 h at 37 °C. With inhibitory halos ranging from 30 to 40 mm for all *Candida* spp. MDR analyzed, group (iv) displayed the greatest results. H_2_O_2_ + lactoferrin-based solutions are thought to be potential PS in PDT for MDR *Candida* spp. eradication.

## 1. Introduction

Lactoferrin (Lf) is a natural iron-binding protein that plays a significant role in the innate immune system, through the production of immunomodulatory proteins, in humans. This enzyme is contained in saliva, breast milk, and some other biological fluids in lower concentrations [1,2]. Salivary Lf also presented an important role in maintaining oral hygiene status. It showed protective function on mucosal surfaces, an important barrier between the host and the external environment, to avoid infections. For these reasons, Lf may be considered a very important protein that is associated with oral mucosal immunity balance [1]. Lf has several antimicrobial properties against bacteria (in particular, against *E. coli* and *P. aeruginosa*), viruses (for example, against HIV, EBV, CMV, SARS CoV-2), and fungi [1,2]. It is also responsible for oxidative stress control in the body, linked to its high affinity for ferric (Feþþþ) ions, and thus, protects cells from oxygen injury [1]. It has been demonstrated that not only the whole protein but also its derived fragments have antimicrobial peptide (AMP) activity, such as Lactoferrampin (Lfampin), and Lf (1–11). The first one comprising residues 268–284 in the N1 domain of Lf, while the Lf (1–11) peptide is a piece of the first 11 amino acid residues of the N-terminal region of Lf and exhibits antimicrobial activity against *C. albicans* and *A. fumigatus* [2]. Lf has a highly cationic N-lobe and can interact with negatively charged cell wall components of Gram-negative and Gram-positive bacteria, producing cell wall destabilization and an increase in membrane permeability, which can determine the cytoplasmic leakage, disruption of balance of solutes and ions within the cell, and ultimately bacterium death [3,4].

### 1.1. Lactoferrin and Oral Disease

*Streptococcus mutans*, the main caries pathogen, *Aggregatibacter actinomycetemcomitans*, *Prevotella*, and *Treponema* spp. are among the oral pathogens against which Lf has demonstrated antibacterial action in in vivo tests [2]. While there is some antimycotic action against *Candida glabrata*, its antifungal effects are primarily directed against *C. tropicalis*, *C. krusei*, and *C. albicans*. Lf’s fungistatic impact is based on its capacity to scavenge iron and activate certain cytokines that are part of the immune response against *Candida* spp., like Th17 [4]. It has, therefore, been suggested as a molecule to be added to prosthesis wearers who are susceptible to denture stomatitis [2]. Additionally, it has been used as an adjuvant to various antifungal medications, including amphotericin B and some azoles, to increase their effectiveness and lower their minimum inhibitory concentration [5]. Combining lactoferrin and lactoperoxidase had even better outcomes in a cell culture investigation on oral *Candida* spp. When compared to other antifungal medications, the two salivary enzymes work together to significantly lower the fungal cell’s size and form, alter its shape, and limit its metabolic activity, demonstrating a potent synergistic effect [6].

### 1.2. Oral Candidiasis

Candida species are the most common fungi found in the oral cavity, although other less common fungal infections can cause oral manifestations, such as mucormycosis, aspergillosis, blastomycosis, histoplasmosis, cryptococcosis, and coccidioidomycosis [7]. Often, oral mucosal infections can be the result of the synergistic action of several types of fungal microorganisms [8]. Candida is a unicellular, dimorphic eukaryotic cell and its reproduction can be sexual or asexual. Outside its cell membrane, there is an outer cell wall. The plasma membrane contains large amounts of ergosterol. The main fungal species are *Candida albicans* (the most common), *C. tropicalis*, *C. glabrata*, *C. krusei*, *C. parapsilosis*, *C. dubliniensis,* and *C. guilliermondii*. In the oral cavity, the *Candida albicans*/*non albicans* species ratio is 68/32%. In patients with candidemia, the association is similar, except for *C. krusei* [9,10]. More than one Candida species can be found in the oral cavity; co-infection with *C*. *albicans* and *C. glabrata* is very common and is associated with a higher likelihood of tissue damage [9]. Some authors have shown that *Candida* spp. may have a mutualistic relationship with other bacteria, i.e., streptococci, in particular, with *S. mitis*, *S. oralis*, *S. gordonii,* and *S. sanguis*. Other researchers have found a co-aggregation between *Candida* spp., *Porphyromonas gingivalis,* and *S. mutans*, favoring the development of periodontal infections and caries [8]. *Lactobacillus* spp., on the other hand, can modulate Candida infection and, therefore, *L. helveticus* and *L. plantarum* have been proposed as probiotics against oral candidiasis [8]. The most common site of adhesion in the oral mucosa is the tongue, but saliva plays a very important role in the spread of this fungus [7]. It has been reported that Candida species can colonize the oral cavity in 45% of neonates, 45–65% of healthy children, 30–45% of healthy adults, and up to 74% of the elderly [7]. Saliva contains several ions, including sodium, potassium, cadmium, chloride, bicarbonate, and phosphate, which are responsible for the buffering properties against *Candida* spp. that grow in an acidic pH. All those conditions that determine a reduced salivary flow or a change in the quality of the saliva predispose to a higher probability of developing candidiasis, such as xerostomia due to radiotherapy, Sjögren’s syndrome, uncompensated diabetes, the presence of oral prostheses, smoking, and poor oral hygiene [7,8,9,10,11,12]. Other conditions that may favor mycotic spread include the prolonged use of medications, especially antibiotics and corticosteroids, debilitating diseases, such as HIV infection, organ transplantation, and cancer. The adhesion of *Candida* spp. is mediated by bacterial components and some mycotic proteins, such as ALS2, ALS3, and others, which are responsible for the transformation into the hyphal form. When the fungus is in this form, it can penetrate the oral mucosal barrier directly through ALS3p anchorage or receptor-induced endocytosis. The presence and rate of candidalysin is the most important factor in epithelial tissue damage [12]. Mucosal integrity, the ability of the immune system to produce interleukins, particularly IL-17, the buffering capacity of saliva, and the bacterial balance may influence oral epithelial adhesion and invasion [12].

One of the most important local factors promoting Candida colonization is the presence of dentures. In adults, between 60% and 100% of denture wearers have a high rate of *Candida* spp. in their oral cavity, which are interesting data when compared to non-wearers [13]. Dentures alter the flow of oxygen and saliva to the underlying tissues, creating an acidic, anaerobic environment that is conducive to Candida growth. Mechanical features of the acrylic prosthesis, such as porosity and hydrophobicity, must be considered as factors favoring Candida infection [14]. As prosthesis wearers are mainly older people, they are certainly the most affected age group in the population. More non-albicans Candida species have been found in 80-year-old patients than in younger patients [15].

Clinical manifestations of oral candidiasis may be acute or chronic [16]. They include pseudomembranous candidiasis, the erythematous form (which includes the subprosthetic form), the atrophic form (which mainly affects the dorsum of the tongue), the chronic hyperplastic form associated with a malignant transformation rate of 12% [17], angular cheilitis often associated with *S. aureus* infection, linear gingival erythema, and median rhomboid glossitis [16].

More recently, oral candidiasis has been associated with potentially malignant lesions (leukoplakia, oral lichen planus, lichenoid) and its role in carcinogenesis has been discovered [18,19,20]. These data highlight the importance of controlling such a common oral infection, even in the prevention of oral cancer.

Recently, a growing and worrying phenomenon has been observed: more and more *Candida* spp. are showing resistance to the most effective antifungal drugs, such as fluconazole, and are, therefore, called multi-drug resistant (MDR). MDR *Candida* spp. are able to enter the bloodstream and cause severe fungal infections that can require hospitalization, intravenous treatments, and even result in death, especially in fragile patients. This phenomenon is a real health emergency [21,22] and researchers are working to find a solution for this type of infection.

### 1.3. Photodynamic Therapy and Lactoferrin

A natural or synthetic photosensitizer that binds selectively to the target cell’s surface, light with a specific wavelength (λ), such as an LED or laser, that activates it, and the bioavailability of oxygen are the three components in photodynamic therapy (PDT), a contemporary non-invasive treatment. The microbial (target) cell is killed when these three components react, producing singlet oxygen and free radicals. The host epithelial cells are thought to be protected [23].

Toluidine blue 1%, methylene blue 0.1–1%, and curcumin-based photosensitizers that are activated, respectively, by light at 630 nm, 660 nm, and 460 nm of λ, are the most commonly PS employed in PDT to treat oral candidiasis; however, they have never been evaluated against MDR *Candida* spp. [24,25].

As an adjuvant to other photosensitizers, including Chlorine6 (Ce6), Lf has been suggested as a catalyst in the treatment of some types of malignant tumors by photodynamic therapy. The mixture with Lf suggested by some authors has demonstrated a significant (>4-fold) reduction in the Ce6 requirement in comparison to free Ce6 in its capacity to trigger light-mediated cell death in carcinoma cells following exposure to light. Even at doses ten times greater than those employed in the PDT study, chlorine6 enriched with Lf has been demonstrated to be non-toxic to cells [26].

Despite its exceptional antimicrobial activity against oral bacteria and fungi, Lf has not yet been suggested as the only photosensitizer in photodynamic therapy for oral infections.

Although there are over a hundred works in the scientific literature on the use of PDT against oral candidiasis, only two works evaluate its efficacy against MDR *Candida* spp. [27,28]. So, we can conclude that there are currently no effective protocols for oral infections by *Candida* spp.

The limitations of the various studies performed on photodynamic therapy for oral candidiasis are linked to the fact that in clinical studies, the size of the areas affected by refractory candidiasis lesions is essentially evaluated, before and after photodynamic therapy, and in vitro the CFU, are often evaluated before and after. These evaluation parameters of the in vivo and in vitro studies are not necessarily an expression of specific efficacy against fungal species. In fact, there is no cut-off to establish what fungal load determines an oral infection [25,26].

One of the problems related to the implementation of PDT in the oral cavity, often in the posterior region and, therefore, adjacent to the hypopharynx, a site often involved in fungal infections, is the possibility of ingesting the photosensitizer and that it is not toxic to the mucosa. This is why the use of lactoferrin could be extremely safe, as a molecule that is already part of the salivary fluid.

Furthermore, lactoferrin, due to its ability to act as a “magnet” for iron, could bind even better to fungal cells than another photosensitizer as they use it for their own survival [3].

Excellent tolerability, the possibility of ingestion of the same, and greater ability to bind precisely with fungal species could be the ideal characteristics of lactoferrin as a photosensitizer.

The aim of this in vitro work is to test a novel natural photosensitizer based on a lactoferrin mixture in PDT against a group of MDR *Candida* spp.

## 2. Materials and Methods

This is a triplicate experiment realized with the Kirby Bauer method using 90 mm diameter Petri dishes for each group of evaluation. For each Petri dishes (three for each group tested) covered with a field of culture, wells of 10 mm were created to put different lactoferrin mixtures inside, through a hot sterilized iron ring. The halo of inhibition after incubation period (48 h) was the expression of the effectiveness of lactoferrin mixtures and the PDT process. The groups for evaluation were 6: 1 control group (without any treatment); 4 experimental groups (i, ii, iii, iv); 1 group treated only with 3% H_2_O_2_, used also in two of the four experimental group as a catalyzer.

### 2.1. Candida spp. Tested

**Figure 1:**
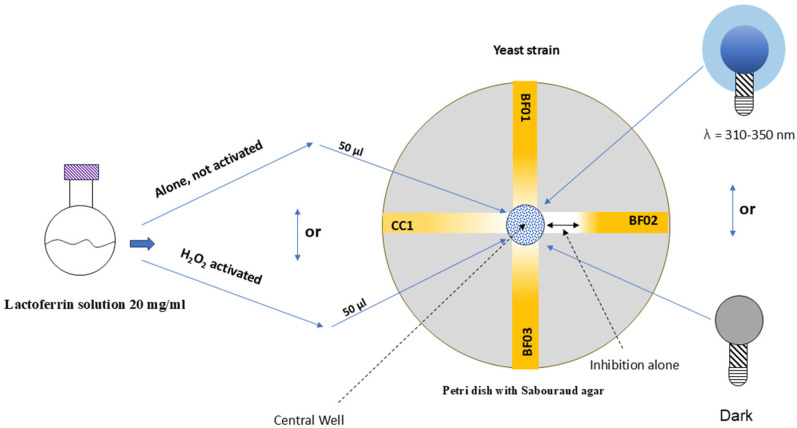
Graphical presentation of Materials and Methods.

Four different clinical isolates of *Candida* spp. were used in this study: *Candida albicans*, BF 01; *Candida krusei*, BF 02; *Candida glabrata*, BF 03 (OBL-Laboratory Collection, Cagliari, Italy); and *Candida albicans* CC1 (which resulted in resistance even to other commercial PDT treatments). In this work, each strain was used in the log growth phase and suspended in Sabouraud dextrose broth (Microbiol, Uta Cagliari, Italy). Then, 1 × 10^7^ CFU/mL of this suspension was radially inoculated on the surface of 90 mm ø Petri dishes containing 15 mL of Sabouraud dextrose agar (Microbiol, Uta Cagliari, Italy). The lactoferrin solution (LF) was put in a well performed inside the agarized medium and located in the center of each dish, 50 μL in each well, Figure 1. Strains BF01, BF02, and BF03 were azole resistant, in particular for fluconazole, voriconazole, and ketoconazole [29].

### 2.2. Lactoferrin Mixture Photosensitizer

A semi-sutured 20 mg of bovine lactoferrin (Sigma Aldrich, Merck KGaA, Darmstadt, Germany) was dissolved in 1 mL of Sabouraud broth and used as the base formula for this in vitro experiment, Figure 1. This concentration value was already described by Li et al. (2019) and Del Olmo et al. (2010) to assess its antitumoral and antimicrobial activity, respectively [30,31]. This mother solution was then used in two different combinations and tested against each strain in the experimental groups: solution as it is; solution activated with 3% H_2_O_2_ (Sigma Aldrich, Merck KGaA, Darmstadt, Germany). The experimental design provided yeast sensitivity measures alone (group i, ii) or under light λ at 310–350 nm (group iii, iv).

The control group was not exposed to lactoferrin solution, 3% H_2_O_2_, or to the LED light (without any type of treatment). It is important to test the real growth of *Candida* spp. MDR tested. The rationale of the group in which *Candida* spp. was exposed only with H_2_O_2_ was to evaluate the H_2_O_2_ impact against *Candida* spp. and to observe the difference of values of inhibition halo with lactoferrin enriched with H_2_O_2_.

### 2.3. Lights to Activate Lactoferrin Mixture

A violet LED light with a wavelength comprised between 310–350 nm of λ, with a power of 400 mWatt (Kireti, Bologna, Italy), was used for the in vitro study. The maximum light absorption for lactoferrin mixture was previously evaluated by a spectrophotometer. The illumination time was 1 min for each Petri dish of (iii) and (iv) experimental groups. An illumination duration of 1 min was chosen because it is in accordance with other works present in the scientific literature, in which similar powers and wavelengths activated the chromophores in in vitro models on plates for 60 s. In addition, 1 min of irradiation represents the medium time indicated in different manufactured instruction for many commercial photodynamic apparatuses.

All Petri dishes were incubated at 37 degrees for 48 h after the PDT experiment and a millimeter gauge was used to evaluate halos of inhibition. For each group and for each *Candida* spp. tested, the average of the values was taken into consideration. The main steps are presented in Figure 1.

### 2.4. Statistical Analysis

All experiments were performed in triplicate. The results were expressed as mean value ± standard deviation. Statistically significant differences among samples were determined using the exact fisher test to substantiate a significant difference between the means of two specific groups. The statistical analysis was performed by using social science statistic software (version 2025, https://www.socscistatistics.com/, accessed on 20 January 2025). The minimum level of significance chosen was *p* < 0.05.

## 3. Results

**Figure 2:**
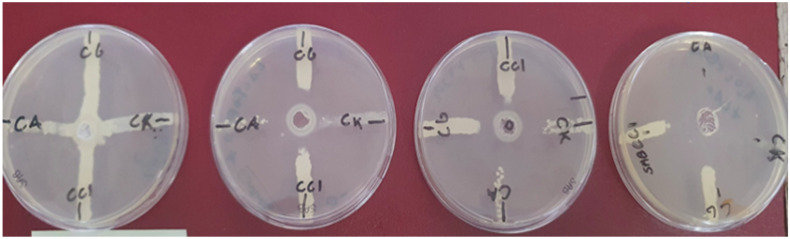
Halos of inhibition against *Candida* spp. MDR evaluated. In figures for each Petri dish: CA = *C. albicans*; CG = *C.glabrata*; CK = *C. krusei;* CC1 = *C.albicans* clinical isolate refractory also to previous PDT. The first Petri dish on the right is one of the three control groups (only *Candida* spp. without any type of treatment with lactoferrin alone or PDT) and the others represent the Petri dish with highest values of inhibition halo found, respectively, from the right to the left, for experimental groups ii, iii, iv (one of the three Petri dishes for each experimental group ii, iii, iv). You can see the clear interruption of the white stripe representing the growth of *Candida* spp. MDR, from the circumference of the well, a tangible sign of an inhibition halo. This is very evident in the iv group where most of the tested fungal colonies were eradicated even a few centimeters from the well containing the photoactivated lactoferrin solution.

All previously identified species of *Candida* spp. are susceptible to the antimicrobial effects of lactoferrin solution, either by itself or in conjunction with light. The lactoferrin mixture evaluated by the Kirby-Bauer test was active alone against *C. glabrata* with a halo of 20 mm, lactoferrin + light (iii group) produced an inhibition halo of 22 mm on *C. albicans*, 30 mm on *C. glabrata*, 20 mm on CC1 (our clinical isolate). Lactoferrin + H_2_O_2_ + light (iv group) produced an inhibition halo of 40 mm on *C. albicans*, 35 mm on *C. glabrata*, 30 mm on *C. krusei*, 30 mm on CC1 clinical isolate. The mean inhibitory halo values with H_2_O_2_ alone were 18 mm on the CC1 clinical isolate, 28 mm on *C. glabrata*, 12 mm on *C. krusei*, and 23 mm on *Candida albicans*. Results are presented in Figure 2 and Table 1.

## 4. Discussion

We discovered some interesting findings regarding the Lf solution as a photosensitizer in the current preliminary in vitro investigation.

The lactoferrin solution was also tested against a clinical isolate of *Candida albicans*, known as CC1, which was extracted from the mouth of a patient who was resistant to nystatin, miconazole, and PDT sessions using 1% toluidine blue activated by 630 nm LED light. In this way, for the first time in the scientific literature, we have tested a particular resistant type of Candida that is refractory not only to pharmacological treatment but also to the photodynamic process with Toluidine Blue 1%, considered one of the most performant PS in PDT against oral candidiasis [25,26].

The lactoferrin mixture alone was active against *C. glabrata* with a halo of 20 mm, lactoferrin + light (310–350 nm) produced an inhibition halo of 22 mm on *C. Albicans*, 30 mm on *C. glabrata*, and 20 mm on CC1 isolate. An inhibitory halo of 40 mm was observed on *Candida albicans*, 35 mm on *Candida glabrata*, 30 mm on *Candida krusei*, and 30 mm on the clinical isolate CC1 by lactoferrin + H_2_O_2_ + light. Mean values of the inhibition halo with H_2_O_2_ alone were 23 mm on *C. albicans*, 28 mm on *C. glabrata*, 12 mm on *C. Krusei*, and 18 mm on CC1 clinical isolate, so we can conclude that the photodynamic activation of lactoferrin solution is not solely caused by the presence of H_2_O_2_ as a catalyst.

Lactoferrin solution showed activity against each type of *Candida* spp. MDR tested. It was also effective against the new clinical isolate CC1, which was the first to emerge from a fungal infection resistant to previous photodynamic treatment. No previous scientific work has evaluated the antifungal activity of a natural product as a photosensitizer on a clinical isolate that is also resistant to previous PDT, which is a novelty.

Although several natural photosensitizers have been proposed, no study has used this type of in vitro experiment, carried out by the Kirby–Bauer method, against *Candida* spp. Therefore, it is not possible to compare our results with those obtained in previous works presented in the literature with other natural photosensitizers, such as curcumin and riboflavin [25]. It is also the first time that a salivary enzyme, often administered to patients in the form of a supplement, has been photoactivated by a photodynamic process, although some authors have hypothesized the use of a lysozyme as an element to be photoactivated in photodynamic therapy [32].

An interesting aspect that emerged from this in vitro study is that the use of H_2_O_2_ to emulsify lactoferrin determines a significant increase in the activity of the photosensitizer, and this could be of interest for the design of other natural photosensitizers to be emulsified or enriched with traces of H_2_O_2_ in future experiments. This is an interesting hypothesis for future works also because the safety of using low concentrations of H_2_O_2_ on oral soft tissues has been well documented in the scientific literature over the last 25 years [33,34,35,36]. The comparison with 3% H_2_O_2_ alone on the inhibition zone shows efficacy results that are far from those of the tested natural products enriched with H_2_O_2_.

The use of violet light in in vivo studies, including in the oral cavity, has been previously documented in the literature [37,38]. In a very recent paper, researchers found that the use of violet diode light does not damage pulp vitality and the results on thermal and pH values are comparable to red light exposure [39]. Previously, 310 and 340 nm light has been successfully used for the treatment of dermatitis and was considered safe [40], so we can conclude that it can also be considered safe when used in the mouth for a few minutes’ activation.

One of the problems related to its use in the oral cavity is the mode of diffusion of the proposed new photosensitizer in the oral cavity. The most appropriate strategy may be to have the patient rinse with a solution of lactoferrin, as described, for a few minutes and then, without rinsing, carry out the illumination in the oral cavity. Indeed, there are mouthwashes on the market based on salivary enzymes including lactoferrin [41], some of them oxygenated [42], which have been used successfully in the treatment of xerostomia, demonstrating that a release model in the oral cavity as hypothesized can be easily achieved.

Delivering lactoferrin through mouthwashes could be a significant challenge for the clinician. In fact, not all people affected by refractory candidiasis and candidates for the hypothetical in vivo PDT treatment are able to retain a mouthwash in their mouth for periods of time sufficient to promote the adhesion of lactoferrin to the mucosa. In fact, these are often elderly patients with poor mobility of the masticatory muscles [7,8,9,10,11,12]. Dissolving orodispersible tablets with lactoferrin in the oral cavity before illumination could be interesting, but it is affected by the salivary flow of the patient candidate for treatment which could be, as is often observed clinically, profoundly altered [7,8]. Another method of delivery could be represented by crumbling a lactoferrin tablet in a gel matrix, such as a product based on sodium carboxymethylcellulose (for exemple Orafix^®^), created specifically to deliver medications in the oral cavity [43,44], where the clinician can decide where to place it exactly.

It remains to be understood what the binding times of lactoferrin to the surface of the cell membranes of the target organisms are, which is an expression of the pre-illumination times.

Although there are other research works on natural photosensitizers, ours is a very original work because of the following: (1) lactoferrin as the only photosensitizer has never been tested in photodynamic therapy in a previous research paper; (2) we have not found other works in which the efficacy of PDT has been evaluated simultaneously on *C. albicans*, *C. glabrata*, *C. krusei,* and a clinical isolate of *C. albicans* not only refractory to drugs but also to a previous PDT session.

In conclusion, this preliminary work shows that a solution based on lactoferrin, enriched or not with H_2_O_2_, could be a new natural photosensitizer, effective in oral refractory infections by *Candida* spp. MDR, when activated by light between 310–350 nm wavelength. Some parameters remain to be evaluated, such as illumination and pre-illumination time on the mucosal surfaces to be treated, or the use of combinations with other adjuvants [45,46,47,48,49,50,51,52], which are expected to be the subject of future randomized clinical trials.

## 5. Conclusions

This in vitro study shows that lactoferrin-based solutions have strong antifungal activity against MDR *Candida* spp., including a clinical isolate that is resistant to earlier pharmacological and PDT treatments. This is especially true when hydrogen peroxide is added, and the solutions are activated by light with a wavelength of 310–350 nm. According to these results, the natural salivary enzyme lactoferrin may be used in PDT procedures as a unique and efficient photosensitizer. The improved effectiveness of H_2_O_2_ emulsification provides fresh insights on how to best optimize natural PS formulations. To specify application conditions and confirm these findings in clinical settings, more investigations are required with a larger sample size.

## Figures and Tables

**Table 1:** Inhibition halo values obtained from lactoferrin-based PDT. For each strain, we indicated the mean values of halo size for each group: control, group only exposed to H_2_O_2_, i, ii, iii, iv experimental groups; the max standard deviation, SD, ranged ±2 mm as inhibition halo diameter.

Strain	Negative Control	Lactoferrin	H_2_O_2_	Lactoferrin + Light	H_2_O_2_ + Light	Lactoferrin H_2_O_2_ + Light
Inhibition halos ø ± 1–2 mm						
*C. albicans*	0	10	23	22	30	40
*C. glabrata*	0	20	28	30	30	35
*C. krusei*	0	10	12	10	30	30
*CC1*	0	10	18	20	18	30

## Data Availability

The original contributions presented in this study are included in the article. Further inquiries can be directed to the corresponding authors.
